# Glutathione and selenium nanoparticles have a synergistic protective effect during cryopreservation of bull semen

**DOI:** 10.3389/fvets.2023.1093274

**Published:** 2023-02-16

**Authors:** Shubin Li, Jingyu Ren, Wenqi Zhang, Biao Wang, Yuzhen Ma, Liya Su, Yanfeng Dai, Gang Liu

**Affiliations:** ^1^Key Laboratory of Medical Cell Biology, Clinical Medicine Research Center, Affiliated Hospital of Inner Mongolia Medical University, Hohhot, Inner Mongolia, China; ^2^College of Life Science, Inner Mongolia University, Hohhot, Inner Mongolia, China; ^3^Animal Husbandry Institute, Inner Mongolia Academy of Agricultural & Animal Husbandry Sciences, Hohhot, Inner Mongolia, China; ^4^Center of Reproductive Medicine, Inner Mongolia People's Hospital, Hohhot, Inner Mongolia, China

**Keywords:** SeNPs, GSH, oxidative stress, bull, semen cryopreservation

## Abstract

**Introduction:**

In the present study, the synergistic protective effect of co-supplementation of glutathione (GSH) with selenium nanoparticles (SeNPs) on the cryopreservation efficiency of bull semen was analyzed.

**Methods:**

After collection, the ejaculates of Holstein bulls were subsequently diluted with a Tris extender buffer supplemented with different concentrations of SeNPs (0, 1, 2, and 4 μg/ml), followed by semen equilibration at 4°C and assessment of sperm viability and motility. Subsequently, the ejaculates of Holstein bulls were pooled, split into four equal groups, and diluted with a Tris extender buffer supplemented with basic extender (negative control group, NC group), 2 μg/ml SeNPs (SeNPs group), 4 mM GSH (GSH group), and 4 mM GSH plus 2 μg/ml SeNPs (GSH + SeNPs group). After cryopreservation, motility, viability, mitochondrial activity, plasma membrane integrity, acrosome integrity, concentration of malondialdehyde (MDA), superoxide dismutase (SOD), and catalase (CAT), and ability of frozen-thawed sperm cells to support *in vitro* embryonic development were evaluated.

**Results and discussion:**

No side effect of SeNPs concentrations applied in the current study on the motility and viability of equilibrated bull spermatozoa was found. Meanwhile, supplementation of SeNPs significantly promoted the motility and viability of equilibrated bull spermatozoa. Furthermore, the co-supplementation of GSH with SeNPs effectively protected bull spermatozoa from cryoinjury as expressed by promoting semen motility, viability, mitochondrial activity, plasma membrane integrity, and acrosome integrity. Finally, the enhanced antioxidant capacity and embryonic development potential in the frozen-thawed bull spermatozoa cryopreserved by co-supplementation of GSH with SeNPs further confirmed the synergistic protective effect of co-supplementation of GSH with SeNPs on the cryopreservation of bull semen.

## Highlights

- 1, 2 and 4 μg/ml SeNPs have no toxic effects on bull spermatozoa.
- Co-supplementation of GSH with SeNPs protects against the cryoinjury of cryopreserved bull spermatozoa.
- Co-supplementation of GSH with SeNPs enhance the ability of frozen-thawed spermatozoa to support *in vitro* development.

## Introduction

The technology of semen cryopreservation effectively promotes the application of technologies including genetic resource conservation, zootechnical management, and artificial insemination (1, 2). In addition, the widely practiced applications of semen cryopreservation significantly enhance the reproductive efficiency and commercial value of livestock breeding (3, 4).

However, the cold shock and alterations in atmospheric oxygen seriously led to biochemical, biophysical, ultrastructural, molecular, and functional alterations (5–7). These alterations in turn trigger excessive production and accumulation of reactive oxygen species (ROS) in mammalian semen (8–11). The imbalance between free radicals and the antioxidant defense system in semen caused by altered ROS production levels led to oxidative stress and osmotic stressors with subsequent acrosomal damage, mitochondrial membrane depolarization, and disruption of plasma membrane permeability (12–16). In addition, oxidative stress-related damage is positively related to genome instability, loss of sperm quality, and fertilization failure (2, 17).

To ameliorate oxidative stress-related damage, the supplementations of antioxidants, including vitamins, coenzymes, exosomes, liposomes, nanomaterials and trace elements such as selenium (Se), copper, and zinc to semen extender have been widely conducted (18–21). As an immune-enhancing micronutrient with excellent antioxidant and anticancer properties, Se plays important role in regulating the mammalian antioxidant system and participates in mammalian spermatogenesis (22). Sperm production and testicular size in Se-deficient rats and mice are seriously disturbed and the stability of sperm mitochondrial sheath is also significantly affected (22). In addition, dietary deficiency of Se inhibits sperm motility and fertility potentials of broiler breeder males (23). These earlier studies found that Se supplementations in the form of selenium nanoparticles (SeNPs) effectively inhibit spermatotoxicity caused by exposure to aflatoxin B1 (24) or cisplatin (25) by reducing spermatic DNA fragmentation and damage. Furthermore, Se supplementations significantly promote the frozen-thawed sperm quality of roosters (21), camels (19), and ram (26). Khalil et al. (27) also found that Se supplementations significantly reduced the chromatin damage in bovine spermatozoa, further convincing the potential applications of SeNPs supplementations in the cryopreservation of mammalian semen. However, these previous studies revealed the low toxicity threshold of Se (>4 μg Se/gm dietary diet), making the application of SeNPs supplementation to the cryopreservation of mammalian semen still needed to be optimized (21, 28, 29).

According to a previous study, the co-supplementation of vitamin E effectively ameliorates the low toxicity threshold of Se (21). However, the potential effect of co-supplementation of glutathione (GSH) with SeNPs on the cryopreservation efficiency of bull spermatozoa has not been reported to date.

GSH, an endogenous antioxidant synthesized from different amino acids including glycine, cysteine, and glutamate, effectively inhibits oxidative stress-related damage and altered ROS production level (30, 31). Our past studies have confirmed the free radicals scavenging potentials of exogenous GSH supplementation on mammalian reproduction potentials (32, 33). Excitingly, exogenous GSH supplementation provides an excellent cryoprotective effect on humans (34), mice (35), jacks (36), canines (37, 38), bulls (39–42), roosters (43–45), ram (46, 47), red deer (48), and boar (49–52) spermatozoa by reducing oxidative stress-related damage. Therefore, it's reasonable to hypothesize that the co-supplementation of GSH with SeNPs would have a synergistic protective effect on the cryopreservation efficiency of bull spermatozoa. This study was designed to investigate the possible synergistic effect of GSH and SeNPs on the post-thawing laboratory indicators of sperm quality, oxidative-antioxidant status, and ability of spermatozoa to support *in vitro* development.

## Materials and methods

### Animals

In this study, five healthy Holstein bulls from Furuijin Animal Husbandry Biotechnology Co., Ltd. (Hohhot, Inner Mongolia) with known fertility were randomly selected for the following experiments (53, 54). The bulls were housed individually in pens and fed under uniform feeding conditions throughout the experiments.

### Chemicals

Unless otherwise indicated, the chemicals, culture medium, and supplements used in this study were purchased from Sigma-Aldrich (Shanghai, China) and Thermo Fisher (Beijing, China). SeNPs with a particle diameter of 40–60 nm were kindly gifted by Mrs. Mengqi Wang from the College of Chemistry and Chemical Engineering at Weifang University and dissolved with the basic semen extender as the working solution.

### Semen collection

For semen collection, bull ejaculates were collected twice a week for 6 weeks with an artificial vagina pre-warmed to 42°C according to the department's protocols (55). Once collected, semen quality parameters in each sample (*n* = 12) were assessed under a phase contrast microscope (CI-L, Nikon, Tokyo, Japan). After the assessment, each bull ejaculate (*n* = 9) with semen volume ≥4 ml, sperm cell concentration ≥1.0 × 10^9^/ml, progressive motility ≥75%, and sperm viability ≥80% were split into aliquots according to the experimental design and applied for the following experiments (27).

### Optimization of SeNPs concentration for semen cryopreservation

The basic semen extender used in this study was a Tris-based extender as distilled water supplemented with 28 mM fructose, 104 mM citric acid, 297 mM Tris buffer, 5.0% (v/v) glycerol, and 20% (v/v) egg yolk.

To optimize SeNPs concentration for subsequent semen cryopreservation, three semen samples were individually split into four equals and diluted to a final concentration of 6.0 × 10^7^ spermatozoa/ml with the basic extender supplemented with 0 μg/ml SeNPs, 1 μg/ml SeNPs, 2 μg/ml SeNPs, and 4 μg/ml SeNPs. Dose selections for SeNPs were conducted according to previous publications with minor modifications (26, 27). Subsequently, diluted semen samples were loaded into 0.25 ml polyvinyl chloride straws, cooled slowly to 4°C in 2 h, and equilibrated at 4°C for a subsequent 2 h. After equilibration, semen samples were collected for the viability and motility assay.

### Semen cryopreservation, drug treatments, and experimental group settings

Before semen cryopreservation, six semen samples were individually split into 4 equals and diluted to a final concentration of 6.0 × 10^7^/ml spermatozoa with the basic extender supplemented with 0 μg/ml SeNPs and 0 mM GSH (negative control group, NC), 2 μg/ml SeNPs (SeNPs group) (27), 4 mM GSH (GSH group) (50) and 4 mM GSH plus 2 μg/ml SeNPs (GSH + SeNPs group). Dose selections for GSH were conducted according to previous publications (5, 56).

For semen cryopreservation, semen samples from each group after dilution were loaded onto semen straws and labeled. After equilibration, the labeled semen straws were inserted into a programmable freezer (IVM Technologies, France). The temperature applied in this study was initially reduced at a programmed rate of −5°C/min from 4°C to −10°C, then at a programmed rate of −40°C/min from −10 to −100°C and finally at a programmed rate of −20°C/min from −100 to −140°C. After cryopreservation, semen straws from each group were immersed in liquid nitrogen for storage.

### Thawing process and quality evaluations of frozen-thawed semen

Two weeks after cryopreservation, frozen straws of each group were picked up and thawed in a 37°C water bath for 30 s (27).

The frozen-thawed sperm motility was visually assessed. Frozen-thawed samples from each group (repeated in triplicated) were placed on glass slides covered with a cover. After the percentage record of linear motile spermatozoa at 200× magnification under a phase contrast microscope in three different microscopic fields for each sample, the final motility ratio of frozen-thawed sperm motility in each group was calculated based on the mean of three successive estimations (27, 55, 57).

The frozen-thawed sperm viability was analyzed by eosin-nigrosin staining (55, 58). Briefly, frozen-thawed samples from each group (repeated in triplicated) were collected and incubated with the eosin-nigrosin solution at 25°C for 2 min and smeared on warm glass slides. The slides were air-dried and examined at 400× magnification under a light microscope. For microscope examination, sperm with unstained sperm were counted as alive and pink-stained sperm were counted as dead. The sperm viability of each group was calculated as the proportion of live spermatozoa/five hundred spermatozoa.

Giemsa staining of frozen-thawed spermatozoa (repeated in triplicated) was performed for the acrosome integrity assay (36). After Giemsa staining at room temperature for 3 h (59, 60), the slides were thoroughly rinsed with distilled water, air-dried, and mounted. For microscope examination at 400× magnification under a phase contrast microscope, 500 spermatozoa per group were classified as viable spermatozoa with intact acrosome stained with pink and non-viable spermatozoa with damaged acrosome stained with dark blue or lavender. The acrosome integrity of different groups was further analyzed.

After incubation with hypo-osmotic swelling solution [HOS, containing 6.75 g/L fructose and 3.67 g/L sodium citrate] at 37°C for 1 h, plasma membrane integrity of each group (repeated in triplicated)was analyzed by evaluating the modifications of sperm tail to score swollen sperm according to previous publications (27, 61).

For mitochondrial function assay, methyl thiazolyl diphenyl-tetrazolium bromide (MTT) assay was performed. Briefly, frozen-thawed spermatozoa from each group (repeated in triplicated) were collected, centrifuged at 500× g for 10 min, and diluted with phosphate buffer saline (PBS) solution. Subsequently, 10 μl MTT solutions were added to 100 μl sperm suspension and incubated at 37°C for 1 h. After MTT incubation, the optical density (OD) value at 550 nm of each group was further recorded with a Multiskan GO spectrophotometer (Thermo Scientific, Beijing, China) (62–65).

### Assessment of antioxidant enzyme activities and oxidative stress markers

To assess antioxidant enzyme activities and oxidative stress markers, frozen-thawed spermatozoa of each group (repeated in triplicated) were collected and centrifuged at 800×g for 20 min to separate spermatozoa from seminal plasma. The collected spermatozoa were washed with PBS solution and centrifuged at 800×g for 20 min. After centrifugation, spermatozoa suspension was sonicated for 10 s on ice repeating six times at intervals of 30 s (66). Subsequently, the homogenate was centrifuged at 800×g for 15 min and the levels of MDA, SOD, and CAT in each group were individually measured by commercial assay kits according to the manufacturer's instructions.

### Oocyte collection and *in vitro* maturation

For *in vitro* maturation (IVM), bovine ovaries, collected from a local slaughterhouse, were kept in normal saline and transported to the laboratory within 2 h. Once in the laboratory, ovaries were thoroughly washed with 70% ethanol and PBS solution to avoid contamination. Subsequently, cumulus-oocyte complexes (COCs) with a diameter of about 100–130 μm and a central oocyte surrounded by at least three layers of cumulus cells were collected according to the department's protocols (32).

After collection, COCs were transferred to 500 μl of IVM droplets (40–50 COCs per droplet) covered by mineral oil and cultured for a subsequent 24 h in a CO_2_ incubator (38.5°C and 5% CO_2_). IVM medium applied in this study was TCM-199 medium supplemented with 0.3 mM sodium pyruvate, 1 μg/ml β-2-oestradiol, 2 mM Gluta^MAX^, 10 ng/ml EGF, 10% fetal bovine serum, 10 U/ml FSH, 10 U/ml LH and 100 μM cysteamine (67–69).

### *In vitro* fertilization and embryonic development analyses

COCs after IVM were collected and transferred to HEPES-synthetic oviductal fluid (H-SOF) medium supplemented with 400 IU/ml hyaluronidase. After incubation at 38°C for 3 min, COCs were gently pipetted to remove surrounding cumulus cells. After microscopic examination, oocytes at metaphase II stages (MII, with the polar body extrusion) were collected and cultured in 500 μl of fertilization droplets as SOF medium supplemented with 5% fetal bovine serum, 2 mM sodium pyruvate, 10 μg/ml heparin sodium and 1 mM caffeine in a CO_2_ incubator (38.5°C, 90%N_2_, 5%CO_2_ and 5%O_2_) (32).

For *in vitro* fertilization (IVF), the frozen-thawed semen of each group was placed in the 15 ml tube containing the Percoll density gradient. After centrifugation at 500×g for 15 min (27, 70), semen samples from each group (*n* = 3) were collected with the number of frozen-thawed spermatozoa adjusted to a final concentration of 1 × 10^6^ sperms/ml, added to the above fertilization droplet containing MII oocytes (repeated in triplicate, 205 oocytes for the NC group, 210 oocytes for the GSH group, 220 oocytes for the SeNPs group and 217 oocytes for the GSH + SeNPs group, repeated in triplicate) and cultured in a CO_2_ incubator (38.5°C, 5%CO_2_ and 5%O_2_) for 18 h. Subsequently, fertilized oocytes from each group were transferred individually to embryo culture droplets as SOF medium supplemented with 0.5 mM sodium citrate, 1 mM sodium pyruvate, 1% non-essential amino acids, 2% essential amino acids, 2 mM Gluta^MAX^, 2.8 mM myo-inositol and 4 mg/ml bovine serum albumin covered by mineral oil in a 35 mm sterile dish, followed by embryo culture in a CO_2_ incubator (38.5°C, 5%O_2_ and 5%CO_2_) (71).

After 48 h, cleaved two-cell embryos from each group were microscopically observed with cleavage rates calculated as the percentage of cleaved two-cell embryos/MII oocytes. Subsequently, embryos of each group were cultured *in vitro* for a further 7 days with the culture medium changed every other day. After embryo culture, the rates of blastocyst development from cleaved two-cell embryos in each group were microscopically observed and calculated as the percentage of blastocysts/cleaved two-cell embryos. Finally, blastocysts from each group were collected and incubated with 5 μg/ml Hoechst 33342 solution at 38°C for 5 min, followed by blastocysts mounting and microscope examination with cell number per blastocyst (*n* = 20 for each group) further recorded.

### Statistical analysis

The experimental data were analyzed by the Statistical Package for the Social Sciences (SPSS, IBM, version 19.0) and determined by parametric (LSD test) and non-parametric (Wilcoxon) tests, based on residual normality (Gaussian distribution) and variance homogeneity. All results were presented as mean ± standard deviation and *P* < 0.05 was considered significant.

## Results

### Effect of SeNPs supplementation on progressive motility and sperm viability of equilibrated bull spermatozoa

**Figure 1:**
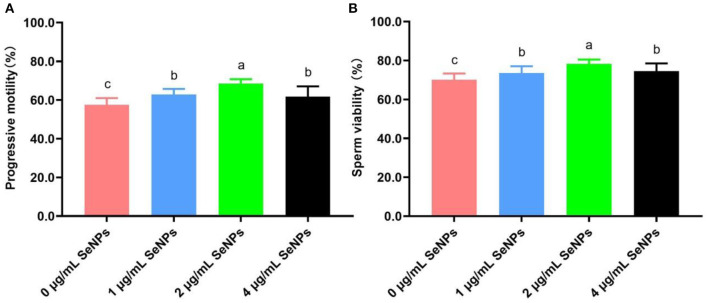
Effect of different concentrations of SeNPs supplementation on motility and viability of equilibrated bull spermatozoa. **(A)** Motility of equilibrated bull spermatozoa. **(B)** Viability of equilibrated bull spermatozoa. Labeling with a different lower-case letter in each column indicates significant differences among groups (*n* = 3, *P* < 0.05).

After the equilibration period at 4°C, progressive motility (62.89 ± 2.85% for the 1 μg/ml SeNPs group, 68.44 ± 2.35% for the 2 μg/ml SeNPs and 61.78 ± 5.29% for the 4 μg/ml SeNPs group, Figure 1A) and viability (73.56 ± 3.47% for the 1 μg/ml SeNPs group, 78.22 ± 2.28% for the 2 μg/ml SeNPs and 74.56 ± 3.97% for the 4 μg/ml SeNPs group, Figure 1B) of equilibrated bull spermatozoa in SeNPs supplementation groups were significantly increased compared to those in the 0 μg/ml SeNPs group (57.56 ± 3.47% for progressive motility and 70.11 ± 3.22% for sperm viability; *P* < 0.05).

In addition, semen motility and viability in the 2 μg/ml SeNPs group were remarkably higher than those in the 1 μg/ml SeNPs and 4 μg/ml SeNPs groups (*P* < 0.05). At the same time, there was a non-statistical difference in progressive motility and sperm viability between the 1 μg/ml SeNPs and 4 μg/ml SeNPs groups (*P* > 0.05).

### Effect of co-supplementation of GSH with SeNPs on quality of cryopreserved bull semen

**Figure 2:**
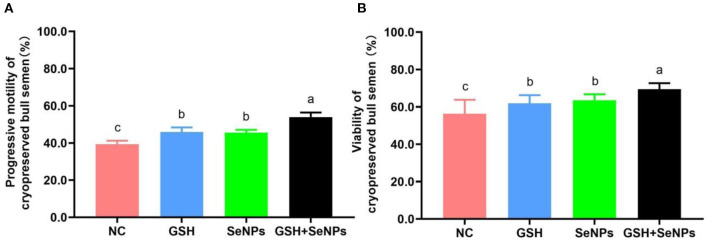
Effect of co-supplementation of GSH and SeNPs on motility and viability of cryopreserved bull spermatozoa. **(A)** Progressive motility of cryopreserved bull spermatozoa. **(B)** Viability of cryopreserved bull spermatozoa. Labeling with a different lowercase letter in each column indicates significant differences among groups (*n* = 6, *P* < 0.05).

As indicated in Figure 2A, the frozen-thawed sperm motility of the GSH + SeNPs group (53.98 ± 2.42%) was significantly higher than that in the NC (39.36 ± 1.86%), GSH (45.87 ± 2.52%) and SeNPs (45.62 ± 1.44%) groups (*P* < 0.05). In addition, there was a non-statistical difference in the post-thaw sperm motility between the GSH and SeNPs groups (*P* > 0.05).

The viability results of frozen-thawed spermatozoa, shown in Figure 2B, were partially consistent with the motility results of frozen-thawed spermatozoa as viability in the GSH + SeNPs group (69.44 ± 3.24%) was significantly higher than that in the NC (56.33 ± 7.43%), GSH (61.89 ± 4.35%) and SeNPs (63.49 ± 3.23%) groups (*P* < 0.05). At the same time, there was a non-significant difference in the motility of frozen-thawed spermatozoa between the GSH and SeNPs groups (*P* > 0.05).

**Figure 3:**
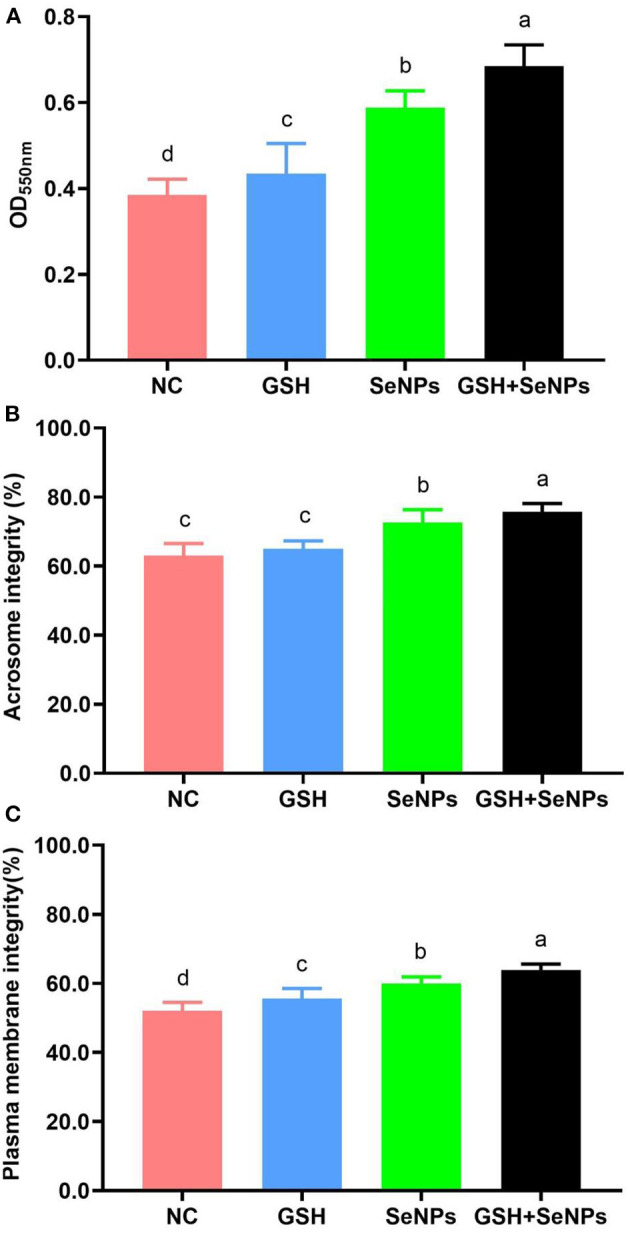
Effect of co-supplementation of GSH and SeNPs on mitochondrial activity, acrosome integrity, and plasma membrane integrity of cryopreserved bull spermatozoa. **(A)** Mitochondrial activity of cryopreserved bull spermatozoa. **(B)** Acrosome integrity of cryopreserved bull spermatozoa. **(C)** Plasma membrane integrity of cryopreserved bull spermatozoa. Labeling with a different lower-case letter in each column indicates significant differences among groups (*n* = 3, *P* < 0.05).

Mitochondrial function assay in this study showed that OD_550nm_ values were remarkably up-regulated from the NC (0.38 ± 0.04), GSH (0.43 ± 0.07), SeNPs (0.59 ± 0.04) groups to the GSH + SeNPs group (0.68 ± 0.05; Figure 3A, *P* < 0.05).

The acrosome integrity of each group was also assessed and the results further confirmed that the acrosome integrity was effectively increased from 63.02 ± 3.52% for the NC group, 65.02 ± 2.25% for the GSH group, 72.58 ± 3.72% for the SeNPs group to 75.67 ± 2.47% for the GSH + SeNPs group (Figure 3B, *P* < 0.05).

Additionally, significant increases in plasma membrane integrity from the NC (52.04 ± 2.46%), GSH (55.67 ± 2.87%), SeNPs (59.89 ± 1.97%) groups to the GSH + SeNPs group (63.82 ± 1.78%) were found in this study (Figure 3C, *P* < 0.05).

### Effect of co-supplementation of GSH with SeNPs on antioxidant potentials of cryopreserved bull spermatozoa

**Figure 4:**
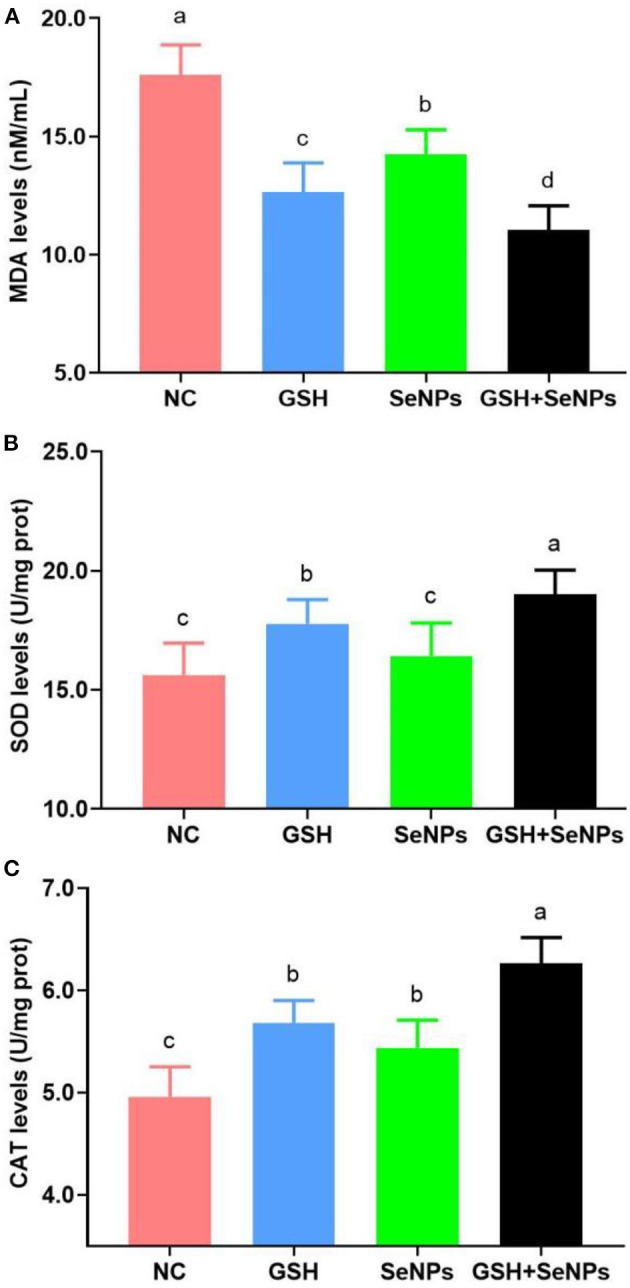
Effect of GSH and SeNPs co-supplementation on antioxidant potentials of cryopreserved bull spermatozoa. **(A)** MDA levels of cryopreserved bull spermatozoa. **(B)** SOD levels of cryopreserved bull spermatozoa. **(C)** CAT levels of cryopreserved bull spermatozoa. Labeling with a different lower-case letter in each column indicates significant differences among groups (*n* = 3, *P* < 0.05).

As shown in Figure 4A, co-supplementation of GSH with SeNPs effectively reduced the MDA levels of frozen-thawed spermatozoa compared to that in the NC group as the MDA levels were effectively reduced from 17.61 ± 1.26 nM/ml for the NC group, 14.23 ± 1.04 nM/ml for the SeNPs group, 12.64 ± 1.23 nM/ml for the GSH group to 11.03 ± 1.03 nM/ml for the GSH + SeNPs group (*P* < 0.05).

Meanwhile, SOD levels in the GSH + SeNPs (19.01 ± 1.01 U/mg prot) and GSH (17.77 ± 1.02 U/mg prot) groups were significantly elevated compared to those in the NC (15.63 ± 1.33 U/mg prot) and SeNPs (16.42 ± 1.39 U/mg prot) groups (Figure 4B, *P* < 0.05). Non-significant differences were found in SOD level between the SeNPs and NC groups (*P* > 0.05). In addition, the SOD level in the GSH + SeNPs group was significantly higher than that in the GSH group (*P* < 0.05).

Results of CAT levels, shown in Figure 4C, confirmed that CAT levels in the GSH + SeNPs (6.26 ± 0.25 U/mg prot), SeNPs (5.44 ± 0.27 U/mg prot), and GSH (5.68 ± 0.22 U/mg prot) groups were significantly increased compared to the NC group (4.96 ± 0.29 U/mg prot, *P* < 0.05). At the same time, a significant increase in CAT level was also found in the GSH + SeNPs compared to the SeNPs and GSH groups (*P* < 0.05).

### Effect of co-supplementation of GSH with SeNPs on development potentials of cryopreserved bull spermatozoa

**Figure 5:**
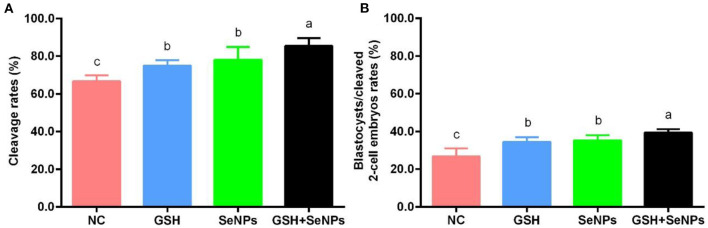
Effect of GSH and SeNPs co-supplementation on cleavage and blastocyst development of cryopreserved bull spermatozoa. **(A)** Cleavage rates of cryopreserved bull spermatozoa. **(B)** Percentage of blastocysts/cleaved 2-cell embryos. Labeling with a different lower-case letter in each column indicates significant differences among groups (*n* = 3, *P* < 0.05).

Embryonic development potentials of frozen-thawed spermatozoa were further analyzed to assess the effect of co-supplementation of GSH with SeNPs on the development potentials of cryopreserved bull spermatozoa with the results present in Figure 5.

After IVF, cleavage rates in the GSH + SeNPs (85.43 ± 4.19%), SeNPs (77.96 ± 6.86%), and GSH (74.94 ± 2.96%) groups were effectively promoted compared to the NC group (66.70 ± 3.06%, Figure 5A, *P* < 0.05). Meanwhile, there was a non-significant difference in cleavage rates between the SeNPs and GSH groups (*P* > 0.05).

Results of blastocyst development rates, shown in Figure 5B, confirmed that the development rate of the blastocyst from cleaved two-cell embryos in the GSH + SeNPs group (39.44 ± 1.80%) was significantly higher than those in the NC (26.67 ± 4.26%), GSH (34.40 ± 2.46%), and SeNPs (35.12 ± 2.77%) groups (*P* < 0.05).

**Figure 6:**
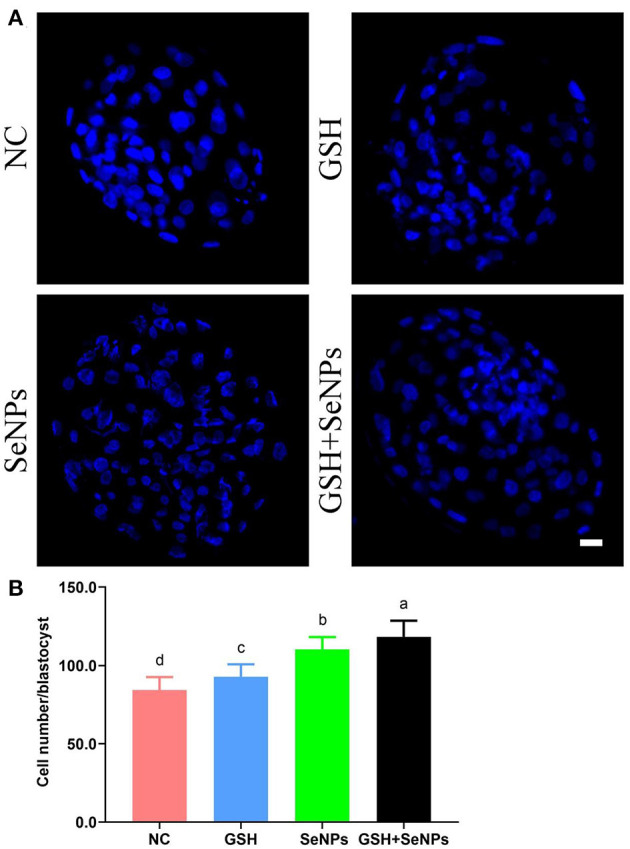
Effect of GSH and SeNPs co-supplementation on blastocyst development of cryopreserved bull spermatozoa. **(A)** Hoechst 33342 staining of the bovine blastocyst. Scale bar = 20 μm. **(B)** Cell number/blastocyst of each group. Labeling with a different lowercase letter in each column indicates significant differences among groups (*n* = 20, *P* < 0.05).

Hoechst 33342 staining of the bovine blastocyst (Figure 6A) was also applied to assess the developmental potentials of cryopreserved bull semen with the cell number/blastocyst of each group analyzed. As indicated in Figure 6B, the cell number/blastocyst in the NC group (84.35 ± 8.29) was significantly lower in comparison with those of the GSH (92.85 ± 7.92), SeNPs (110.30 ± 7.79), and GSH + SeNPs (118.25 ± 10.37) groups (*P* < 0.05). Meanwhile, the cell number/blastocyst in the GSH + SeNPs group was significantly higher than those of the SeNPs and GSH groups (*P* < 0.05).

## Discussion

In this study, we found that co-supplementation of GSH with SeNPs has a synergistic protective effect, which is manifested up-regulation of semen motility, viability, mitochondrial activity, plasma membrane integrity, acrosome integrity, antioxidant penitential, and embryonic development.

The promising effect of SeNPs supplementation on the cryopreservation efficiency of bull semen was confirmed in the present study, partially consistent with Khalil's findings (27). Khalil et al. (27) found that the supplementation of commercial SeNPs (with a particle diameter of 87.6 ± 5.81 nm) to the bull semen extender at a level of 0.5 or 1.0 μg/ml promoted motility, viability, and plasma membrane integrity of bull semen with the levels of sperm abnormality and chromatin damage reduced. They also confirmed that excessive supplementation with SeNPs (>1.5 μg/ml) decreased the viability of bull semen and increased the apoptotic rates of bull semen. Meanwhile, the toxicity of 1.5 μg/ml SeNPs supplementation on bull semen was correlated with decreased activity of glutathione peroxidase and increased level of lipid peroxidation, which enhanced the apoptosis of bull semen. To avoid the toxicity of overdose SeNPs supplementation, different concentrations of SeNPs supplementation to the bull semen extender were analyzed in this study. We found that after equilibration, motility and viability of bull spermatozoa in the 2 μg/ml SeNPs group were increased compared to the 0 μg/ml SeNPs, 1 μg/ml SeNPs, and 4 μg/ml SeNPs groups. Our study additionally confirmed the beneficial effect of 2 μg/ml SeNPs supplementation on the effectiveness of cryopreservation of bull semen. Combined with the embryonic developmental potential result of frozen-thawed bull spermatozoa, our study demonstrated the non-existent toxicity of 2 μg/ml SeNPs supplementation on cryopreservation of bull semen. We hypothesized that the reason for the different results compared with Khalil's study may be due to the particle size and component of SeNPs. Accumulating studies have pointed out that the modifications of pore size, particle size, surface charge, and mechanical strength of biomaterials influence their bioactivities and mechanical properties (72–74). However, the bioactivities of SeNPs need further investigations to confirm their bioactivities and application potentials in the cryopreservation of bull semen.

In addition, we found that the co-supplementation of 4 mM GSH with 2 μg/ml SeNPs to the bull semen extender promoted the cryopreservation efficiency of bull semen. El-Batal et al. (75) found that the synthesized incorporation of GSH and SeNPs promoted the antimicrobial activity, anticancer activity, and high DPPH scavenging ability of GSH or selenium dioxide, partly consistent with our findings. We assumed that the synergistic protective effect of co-supplementation of GSH with SeNPs on the cryopreservation efficiency of bull semen may be due to the particular characteristic of nanomaterials on enhancing the antioxidant potentials of GSH (75), which needs further detailed research.

Our former studies have confirmed that GSH supplementation promoted mitochondrial functions of mammalian gametes (32, 33). These former publications have confirmed the beneficial effect of co-supplementation of GSH with BIOXcell extender, raffinose, sucrose, and low-density lipoproteins on the cryopreservation of bull semen (40, 54, 76–79). Our study also confirmed that GSH supplementation promoted motility, viability, plasma membrane integrity, mitochondrial activity, antioxidant ability, and embryonic development of cryopreserved bull spermatozoa. However, the acrosome integrity of frozen-thawed bull spermatozoa was not affected by the GSH supplementation alone. We hypothesize that controversial results about the effectiveness of GSH supplementation alone on the cryopreservation of mammalian semen may be due to the differences in species, extender composition, and GSH concentration (5, 53, 80–82), which still need further detailed investigations.

Moreover, the current study was limited by the lacking of DNA integrity and *in vivo* fertility assessment to confirm the synergistic protective effect of co-supplementation of GSH with SeNPs on the cryopreservation efficiency of bull semen. We hope that the limitations of the present study will warrant more research to determine the potential effect of co-supplementation of GSH with SeNPs on the cryopreservation efficiency of bull semen.

In conclusion, the co-supplementation of 4 mM GSH with 2 μg/ml SeNPs significantly promoted motility, viability, mitochondrial activity, plasma membrane integrity, acrosome integrity, antioxidant ability, and embryonic development potentials of cryopreserved bull spermatozoa. We sincerely hope that the finding in the present study would benefit the promising applications of co-supplementation of GSH with SeNPs in the future applications of bull semen cryopreservation.

## Data availability statement

The original contributions presented in the study are included in the article/supplementary material, further inquiries can be directed to the corresponding author.

## Ethics statement

The animal study was reviewed and approved by the Animal Care Committee of Inner Mongolia University. Written informed consent was obtained from the owners for the participation of their animals in this study.

## Author contributions

Data curation, formal analysis, project administration, supervision, writing—original draft, and writing—review and editing: GL. Funding acquisition: BW, YM, LS, and YD. Investigation: SL, JR, WZ, BW, YM, LS, YD, and GL. Methodology: GL and YD. Resources: YD. All authors have read and approved the final manuscript.
